# Differential *SP4* expression and HSP60 abundance in buccal swabs from patients with schizophrenia

**DOI:** 10.1126/sciadv.aeb0460

**Published:** 2026-03-04

**Authors:** Christen M. Crosta, Brandon J. Vaglio, Joshua Stuckey, Atul K. Bhattiprolu, Johanne Solis, Jared Lamp, Andrew Umstead, Irving E. Vega, Steven M. Silverstein, Bonnie L. Firestein

**Affiliations:** ^1^ Department of Cell Biology and Neuroscience, Rutgers, The State University of New Jersey, Piscataway, NJ 08854, USA.; ^2^ Neurosciences Graduate Program, Rutgers, The State University of New Jersey, Piscataway, NJ 08854, USA.; ^3^ Biomedical Engineering Program, Rutgers, The State University of New Jersey, Piscataway, NJ 08854, USA.; ^4^ Brain Health Institute, Rutgers, The State University of New Jersey, Piscataway, NJ 08854, USA.; ^5^ Integrated Mass Spectrometry Unit, Department of Translational Neuroscience, College of Human Medicine, Michigan State University, Grand Rapids, MI 49503, USA.; ^6^ Department of Psychiatry, University of Rochester Medical Center, Rochester, NY 14642, USA.

## Abstract

Schizophrenia (SCZ) is heterogenous and polygenic, making it difficult to diagnose and treat. This is expected as diagnostic criteria are solely based on behavioral markers. There is a critical need for easy-to-collect biomarkers that aid in treatment. To identify potential biomarkers, we recruited patients with SCZ and controls. Using buccal cell lysates, we performed real-time quantitative polymerase chain reaction and identified significant differences in *Sp4* mRNA between patients and controls. Targeted mass spectrometry identified increased heat shock protein 60 (HSP60) in SCZ samples. To determine the utility of *Sp4* mRNA and HSP60 protein as biomarkers, we evaluated their relationship with symptom severity and aberrant cognitive processes. Correlational analyses revealed that elevated *Sp4* mRNA and HSP60 protein define a subgroup of patients with SCZ who demonstrate symptomology and poor memory. These data support buccal cell *Sp4* mRNA and HSP60 protein as easy-to-collect, candidate SCZ biomarkers for a subset of patients.

## Teaser

Buccal cell biomarkers have the potential for diagnosis and treatment of schizophrenia.

## INTRODUCTION

Schizophrenia (SCZ) is a debilitating and chronic psychiatric illness characterized by three clusters of symptoms: positive, negative, and cognitive (*1*, *2*). Positive symptoms describe experiences present in patients that are absent in healthy individuals, such as delusions and hallucinations (*2*). Negative symptoms describe behaviors and experiences absent in patients but present in healthy populations, including flat affect, avolition, and social withdrawal (*2*). Cognitive symptoms describe deficits in a range of cognitive functions, including attention, memory, abstraction, problem solving, and executive functioning (*2*). Negative and cognitive symptoms are particularly difficult to ameliorate with current medications, yet they contribute substantially to disability (*1*, *2*).

SCZ has an estimated global age-standardized point prevalence of 0.28% (*3*). Despite this low prevalence, SCZ contributes substantially to the global burden of disease, resulting in 13.4 million years of life lived with disability (*3*). Moreover, only ~13.5% of patients with SCZ recover, based on clinical and social recovery indices (*3*). Many do not respond well to current treatments, and common outcomes include poor quality of life, social disability, long-term hospitalization, suicide attempts, and premature mortality (*4*). Despite a two- to threefold increased risk of death for individuals with SCZ, disease etiology is still poorly understood (*1*, *5*, *6*). This is predominately because SCZ is highly heterogeneous and polygenic, making it particularly challenging to identify the genetic causes of the disease (*6*).

Currently, psychiatrists rely on diagnostic frameworks described in the Diagnostic and Statistical Manual of Mental Disorders (DSM-5) to make categorical diagnoses based on behavioral phenotypes. Molecular and clinical neuroscience findings are not considered (*7*, *8*). Thus, it is likely that diagnostic categories for SCZ include individuals with multiple and different underlying pathophysiologies (*9*). Psychiatric medications are prescribed in a trial-and-error fashion, and identification of an effective medication regimen, when one can be identified, can take up to a year. Medications are prescribed to treat SCZ symptoms, not the underlying biological mechanisms (*8*). Current treatments for SCZ only alleviate a subset of disease symptoms and have several undesirable side effects (*2*, *7*, *9*), resulting in low treatment compliance among patients (*2*, *10*, *11*).

To improve the diagnosis and treatment of SCZ, it is imperative to identify relevant biomarkers (*12*). Biomarkers are useful for diagnostic purposes, monitoring disease progression, increasing therapeutic efficacy, and understanding biological mechanisms. Genetic markers, blood- and tissue-derived markers (e.g., protein levels), electroencephalographic signatures, and neuroimaging and neuropsychological task–based markers, are essential for understanding the pathophysiology of psychiatric diseases (*7*). However, there are no standard biomarkers used to diagnose and aid in the treatment of patients with SCZ.

Here, we sought to identify potential biomarkers for SCZ in buccal swabs that define a subgroup of patients based on symptomology and poor performance on cognitive slowing and memory tests. We recruited patients with SCZ and age-, race-, and gender-matched control (CTRL) subjects. Buccal cells were chosen for our study because the central nervous system (CNS) and buccal epithelium both develop from embryonic ectodermal tissue, i.e., neural and surface ectoderm, respectively, and the CNS and buccal cells influence each other during development (*13*). Furthermore, genetic changes during development can affect both neurons and oral and facial structures as these are influenced by the neural crest (*14*). Buccal swabs are time-efficient and easy to acquire (*15*). We began by studying SP4 (specificity protein 4, Sp4), NOS1AP (nitric oxide synthase 1 adaptor protein), and RASD1 (encodes dexamethasone-induced ras-related protein 1; Dexras1) as possible buccal cell biomarkers for SCZ. SP4 and NOS1AP are encoded by SCZ-risk genes (*16*–*22*) and expressed outside the CNS (*23*–*28*). RASD1, or Dexras1, is a binding partner of NOS1AP (*21*, *29*, *30*) and is also expressed in somatic cells (*31*, *32*).

We found that patients with SCZ have significantly greater levels of buccal cell *Sp4* mRNA than do CTRL subjects. Increased *Sp4*, but not *NOS1AP* or *Dexras1*, mRNA transcripts are associated with poorer performance on the Revised Hopkins Verbal Learning Test (HVLT-R), specifically the recognition portion. Furthermore, higher *Sp4* expression correlates with increased presence and severity of positive and negative symptoms and excitement. Proteomic analysis of buccal cell extracts revealed significant protein abundance differences between patients with SCZ and CTRL subjects in several cellular pathways, including neutrophil degranulation, eukaryotic translation, mitochondrial dysfunction, and oxidative phosphorylation. Differences in abundance were seen between proteins that are targets of SP4. In particular, patients with SCZ had higher protein abundance of heat shock protein 60 (HSP60), and we observed that higher HSP60 abundance significantly correlates with poor performance on cognitive tasks. HSP60 is encoded by an SCZ-risk gene, and it is a predicted target of SP4 as its proximal promoter contains two GC boxes (*16*, *33*, *34*). Together, our data suggest that *Sp4* mRNA transcript and HSP60 protein abundance are biomarkers of SCZ, are easy to collect from buccal cells, and are associated with SCZ-related cognitive dysfunction, helping to define a subset of patients with SCZ.

## RESULTS

### Participant demographics

We recruited participants from the surrounding area to Rutgers University. Patients were 18 to 65 years of age and had a diagnosis of SCZ or schizoaffective disorder. All diagnoses were confirmed by the Structured Clinical Interview for DSM-5 (SCID-5) and in consultation with a psychiatrist at the Rutgers-Princeton Center for Computational Cognitive Neuropsychiatry (CCNP). We recruited CTRL subjects with similar anthropometric data (+/− 6 years of age, race, gender) to patients with SCZ to maintain equivalent demographics between the groups with *n* = 27 for each group (*35*). Demographic characteristics are shown in Table 1.

**Table 1.** Participant demographics. Demographic data for patients, age-, race-, and gender-matched CTRL subjects, and aggregated samples.

Variable		SCZ	CTRL	Total
**Sex**		*n*, %	*n*, %	*n*, %
	Male	17, 63%	17, 63%	34, 63%
	Female	10, 37%	10, 37%	20, 37%
**Race**				
	Asian	7, 25.9%	7, 25.9%	14, 25.9%
	Black	14, 51.9%	13, 48.1%	27, 50.0%
	White	6, 22.2%	7, 25.9%	13, 24.1%
**Ethnicity**				
	Hispanic/Latino	7, 25.9%	7, 25.9%	14, 25.9%
	Not Hispanic/Latino	20, 74.1%	20, 74.1%	40, 74.1%
**Age**		M (SD)	M (SD)	M (SD)
		37.89 (15.56)	37.81 (15.51)	37.85 (15.38)

### Patients with SCZ have significantly higher levels of *Sp4 mRNA* in buccal cell swabs compared to that of CTRL

**Fig. 1.**
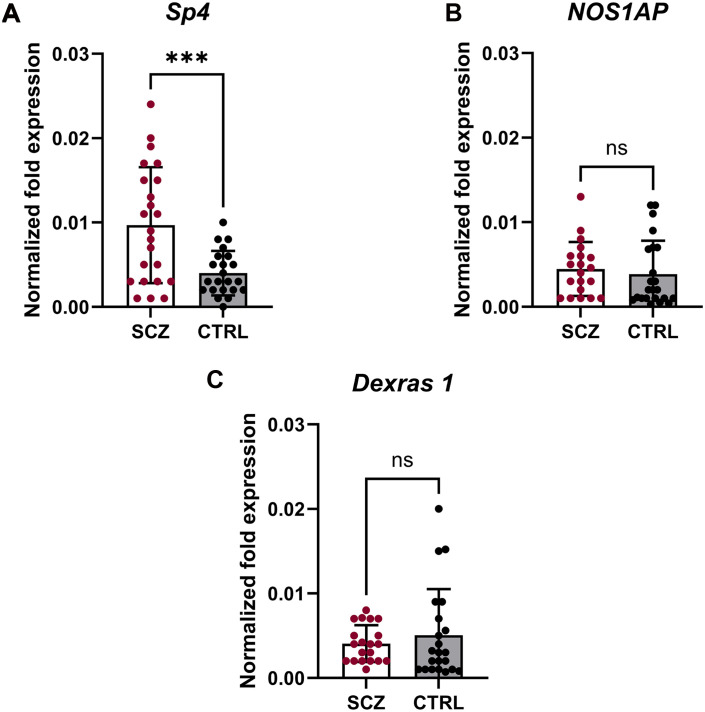
Expression of *Sp4*, but not *NOS1AP* or *Dexras1*, mRNA is increased in buccal cells of patients with SCZ. (**A**) RT-qPCR analysis of *Sp4* mRNA [normalized to glyceraldehyde-3-phosphate dehydrogenase (*GAPDH*)] in buccal cell extracts from patients with SCZ (*n* = 23) or CTRL subjects (*n* = 22). ***P = 0.0009 as determined by unpaired *t* test with Welch’s correction for unequal variances. (**B**) RT-qPCR analysis of *NOS1AP* mRNA (normalized to *GAPDH*) in buccal cell extracts from patients with SCZ (*n* = 20) or CTRL subjects (*n* = 23). No changes were observed (*P* = 0.59) as determined by unpaired *t* test. ns, not significant. (**C**) RT-qPCR analysis of *Dexras1* mRNA (normalized to *GAPDH*) in buccal cell extracts from patients with SCZ (*n* = 20) or CTRL subjects (*n* = 22). No changes were observed (*P* = 0.43) as determined by an unpaired *t* test with Welch’s correction for unequal variances. Error bars represent SD. For all analyses, outliers were removed [(A) = 4, (B) = 6, and (C) = 9] using the ROUT method (*Q* = 1%), and samples with threshold cycle (Ct) values below detection levels or without an age-, race-, and gender-matched CTRL were removed.

We selected proteins encoded by two SCZ-risk genes—*Sp4* (Sp4) (*16*, *17*, *19*, *22*) and *NOS1AP* (*18*, *20*, *21*)—and a binding partner of NOS1AP and *RASD1* [encodes Dexras1 (*21*, *29*, *30*)] as possible buccal cell biomarkers. We performed reverse transcription quantitative polymerase chain reaction (RT-qPCR) to determine mRNA expression levels of our genes of interest in both patients and matched CTRLs. We found that patients with SCZ had significantly higher buccal cell *Sp4* mRNA expression than CTRL subjects (*P* < 0.001) (Fig. 1A). However, no such differences were observed for *NOS1AP* (*P* = 0.59) or *RASD1* (*P* = 0.43) (Fig. 1, B and C). These findings were verified using an orthogonal *SP4* primer set (fig. S2A) and replicated using the original primer set (fig. S2B). These data indicate that *Sp4* mRNA up-regulation is associated with SCZ, which may serve as potential diagnostic biomarker.

### Higher *Sp4* expression in buccal cell swabs is associated with slower response times in the AX-CPT

**Fig. 2.**
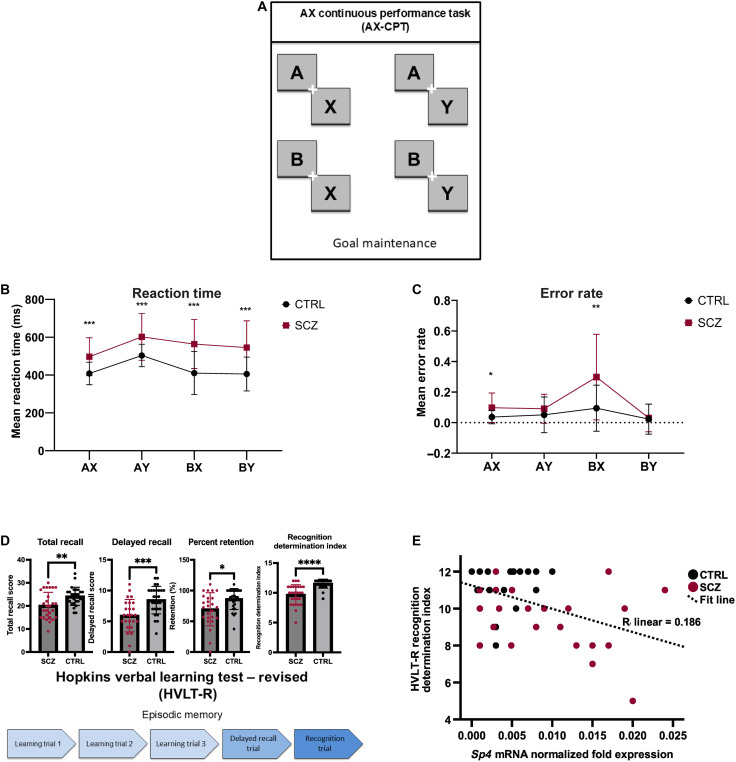
*Sp4* mRNA expression in buccal cells correlates poorer performance on the recognition portion of the HVLT-R. (**A**) Schematic of the four trial types in the AX-CPT task, with AX being the target. (**B**) Mean reaction time by trial type. Multiple unpaired *t* tests with Welch’s correction were used to determine differences by trial type. Adjusted *P* values were calculated using the Holm-Šídák method. Significant differences in reaction times were seen across all trial types (*P* < 0.001). (**C**) Mean error rate by trial type. Multiple unpaired *t* tests with Welch’s correction were used to determine differences by trial type. Adjusted *P* values were calculated using the Holm-Šídák method. No significant differences between patients with SCZ and CTRL subjects were observed for the AY (*P* = 0.33) and BY (*P* = 0.79) trials. However, patients with SCZ had significantly higher mean error rates in the AX (*P* = 0.02) and BX (*P* = 0.01) trials. (**D**) Schematic of trial progression in the HVLT-R. Adapted image from “Timeline (Layout 5 × 1)” in BioRender templates (freely available) and drawn in Powerpoint. Unpaired *t* tests were used to determine *P* values; Welch’s correction was used in cases of unequal variance. Outliers (recognition *n* = 1) were removed using the ROUT method (*Q* = 1%). Patients with SCZ scored lower in the categories of total recall (*P* = 0.004), delayed recall (*P* = 0.0005), retention (*P* = 0.012), and recognition (*P* < 0.0001). Error bars represent SD. (**E**) Increased *Sp4* expression significantly correlates with lower recognition determination scores in the HVLT-R task as determined by Pearson’s *r*, two-tailed [*r*(45) = −0.431, *P* = 0.012]. *P < 0.05, **P < 0.01, ***P < 0.001, ****P < 0.0001.

To determine whether changes to *Sp4* mRNA levels correlate with cognitive impairment, we administered clinical structured interviews and a battery of tasks that are sensitive to the cognitive deficits characteristic of patients with SCZ. We tested our subjects using the AX continuous performance task (AX-CPT) and jittered-orientation visual integration (JOVI) task, which measure goal maintenance and cognitive control (*36*) and visual perception (*37*), respectively. In all trial types of the AX-CPT (Fig. 2A), CTRL subjects demonstrated significantly faster performance than their SCZ counterparts (*P* < 0.001) (Fig. 2B). Similarly, on the AX and BX trials, patients with SCZ were significantly less accurate in their responses (*P* < 0.05) (Fig. 2C). On the JOVI task, CTRL subjects had significantly faster performance on the 0° jitter condition and both catch conditions (*P* < 0.05) when compared to patients with SCZ (fig. S1B). Moreover, in the 7°, 9°, and 11° conditions of the JOVI, patients with SCZ made significantly more errors than CTRL groups (*P* < 0.05) (fig. S1C). Correlation analyses between *Sp4* mRNA and measurements of accuracy and reaction time in both the AX-CPT and JOVI task failed to show significant correlations (table S4). In a series of multiple linear regression analyses, we examined the ability of *Sp4* mRNA to predict performance on the AX-CPT and JOVI tasks (table S5). Covariates (age, sex, ethnicity, and race) entered at step 1 accounted for a nonsignificant amount of variance in AX-CPT BX trial reaction time [adjusted coefficient of determination (*R*^2^) = −0.012; *P* = 0.490], whereas addition of *Sp4* mRNA at Step 2 accounted for a significant amount of additional variance (adjusted *R*^2^ = 0.185; *P* = 0.024) (table S5). This is the only finding that remained significant after controlling for multiple comparisons. These data suggest that *Sp4* mRNA expression in buccal cells is associated with cognitive slowing on a working memory task but not with visual integration deficits.

### Higher buccal cell *Sp4* expression correlates with poorer performance on the recognition portion of the HVLT-R

To determine whether buccal cell *Sp4* mRNA expression can serve as a biomarker for memory dysfunction in SCZ, we used the HVLT-R performance task. Overall, we found that patients with SCZ demonstrate deficits in HVLT-R task performance. Specifically, patients scored lower in the categories of total recall, delayed recall, retention, and recognition (*P* < 0.05) (Fig. 2D). We then explored the relationship between HVLT-R performance and *Sp4* mRNA expression and found a statistically significant, moderate negative correlation between *Sp4* mRNA expression and recognition determination in the HVLT-R task [*r*(45) = −0.431, *P* = 0.012] (table S4 and Fig. 2E). Multiple linear regression analysis corroborated that *Sp4* mRNA can predict recognition performance on the HVLT-R. Covariates (age, sex, ethnicity, and race) accounted for a nonsignificant amount of variance in recognition determination (adjusted *R*^2^ = −0.024; *P* = 0.572), whereas the addition of *Sp4* mRNA to the model accounted for a significant amount of additional variance (adjusted *R*^2^ = 0.129; *P* = 0.028) (table S5). The significant relationships that are reported here are those that remained significant after correction for multiple comparisons. Thus, higher *Sp4* expression correlates with lower recognition, but not recall performance, suggesting a relationship between increased *Sp4* mRNA and information encoding and/or storage impairments (tables S4 and S5).

### Higher *Sp4* expression in buccal cell swabs correlates with increased severity of symptoms

**Fig. 3.**
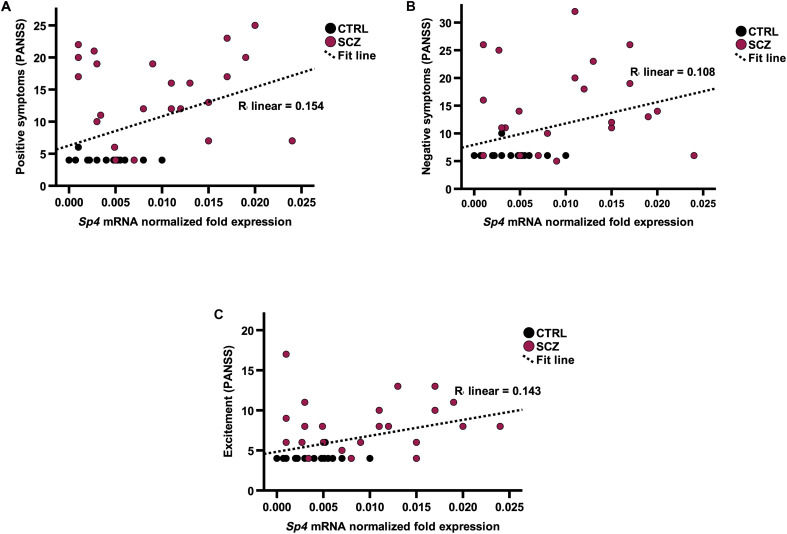
*Sp4* mRNA expression in buccal cells significantly correlates with symptom severity as measured by the PANSS five-factor model. (**A** to **C**) Increased *Sp4* expression significantly correlates with an increased presence of positive (A), negative (B) symptoms, and excitement (C) as determined by Pearson’s *r*, two-tailed [*r*(45) = 0.393, *P* = 0.026; *r*(45) = 0.329, *P* = 0.046; *r*(45) = 0.379, *P* = 0.026, respectively].

Since patients with SCZ experience defined symptoms, we evaluated whether buccal *Sp4* mRNA expression levels correlate with the presence of these different symptoms using the SCI–positive and negative syndrome scale (PANSS). On the basis of a five-factor and cluster scoring solution, our SCZ cohort ranged from moderately to markedly ill across factors (Table 2) (*38*–*40*). We found a statistically significant, moderate positive correlation between *Sp4* mRNA expression and the severity of positive [*r*(45) = 0.393, *P* = 0.026] and negative [*r*(45) = 0.329, *P* = 0.046] symptoms (table S4 and Fig. 3, A and B). Moreover, we also observed significant relationships between *Sp4* mRNA expression and the severity of excitement [*r*(45) = 0.379, *P* = 0.026] (table S4 and Fig. 3C). A series of multiple linear regression analyses corroborated these results (table S5). We found that while age, sex, ethnicity, and race accounted for a nonsignificant amount of variance in positive (step 1 adjusted *R*^2^ = 0.023; *P* = 0.750), negative (step 1 adjusted *R*^2^ = −0.043; *P* = 0.835), and excitement (step 1 adjusted *R*^2^ = −0.023; *P* = 0.835) scores on the PANSS, the addition of *Sp4* mRNA accounted for a significant amount of additional variance for positive (step 2 adjusted *R*^2^ = 0.168; *P* = 0.018), and excitement (step adjusted 2 *R*^2^ = 0.137; *P* = 0.018), but not for negative (step 2 adjusted *R*^2^ = 0.054; *P* = 0.050). The significant relationships that are reported here are those that remained significant after correction for multiple comparisons. These data give additional support for buccal cell *Sp4* mRNA as a potential biomarker related to the clinical expression of SCZ.

**Table 2.** PANSS scores. PANSS scores for both groups using five-factor and cluster scoring. *P* values determined using unpaired *t*-test with Welch’s correction for unequal variances.

Variable	SCZ	CTRL	*P* value
PANSS: Positive symptoms, mean (SD)	15.04 (6.49)	4.26 (0.764)	<0.001
PANSS: Negative symptoms, mean (SD)	15.48 (7.27)	6.26 (0.944)	<0.001
PANSS: Cognitive symptoms, mean (SD)	11.04 (3.88)	5.19 (0.557)	<0.001
PANSS: Excitement, mean (SD)	9.15 (4.63)	4.07 (0.385)	<0.001
PANSS: Depression, mean (SD)	13.44 (6.14)	5.67 (2.35)	<0.001
Cuesta and Peralta PANSS: Disorganization, mean (SD)	6.19 (3.25)	3.04 (0.192)	<0.001
Cluster scoring for PANSS: Anergia, mean (SD)	7.78 (4.57)	4.00 (0.00)	<0.001
Cluster scoring for PANSS: Thought disturbance, mean (SD)	13.15 (5.81)	4.15 (0.534)	<0.001
Cluster scoring for PANSS: Activation, mean (SD)	5.96 (2.74)	3.00 (0.00)	<0.001
Cluster scoring for PANSS: Paranoid/belligerence, mean (SD)	7.70 (3.00)	3.19 (0.681)	<0.001
Cluster scoring for PANSS: Depression, mean (SD)	11.93 (5.58)	4.22 (0.641)	<0.001

### Mitochondrial and immune pathways are altered in buccal cells in patients

**Fig. 4.**
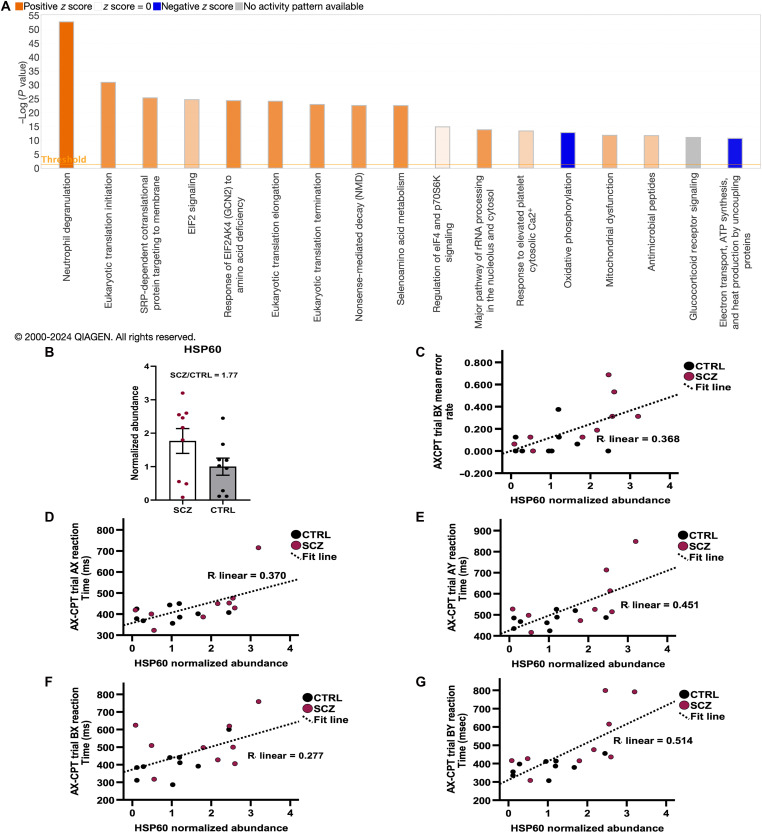
HSP60 abundance in buccal swabs correlates with poor AX-CPT performance. Label-free quantitation of buccal swab proteomics reveals differences in the abundance of mitochondrial, immune, and metabolism associated proteins in SCZ subjects versus CTRL subjects. (**A**) Relevant canonical pathways with the greatest differences in buccal cells from patients with SCZ versus CTRL subjects. Orange indicates a positive *z* score, predicted pathway activation; blue indicates a negative *z* score, predicted pathway inhibition; gray indicates that Ingenuity Pathway Analysis (IPA) was unable to predict a pattern of activity. Opacity indicates the degree of predicted activation or inhibition. Data were analyzed, and graph was created with the use of QIAGEN IPA (QIAGEN Inc., https://digitalinsights.qiagen.com/IPA). (**B**) HSP60 is in greater abundance in buccal cells from patients with SCZ (protein abundance of each patient sample normalized to the average HSP60 abundance of the CTRLs; fold change = 1.77). Error bars represent SD. (**C**) Increased HSP60 abundance significantly correlates with BX error rate on the AX-CPT as determined by Pearson’s *r*, two-tailed [*r*(18) = 0.606, *P* = 0.015]. (**D** to **G**) HSP60 abundance significantly correlates with slower reaction time in all trial types of the AX-CPT using Pearson’s *r*, two-tailed [AX: *r*(18) = 0.608, *P* = 0.015; AY: *r*(18) = 0.671, *P* = 0.009; BX: *r*(18) = 0.527, *P* = 0.040; BY: *r*(18) = 0.717, *P* = 0.007].

Since SP4 is a transcription factor (*23*), we determined whether any of its downstream targets could also be used as a biomarker for SCZ. We reasoned that changes to *Sp4* mRNA levels could reflect increased SP4 protein levels and, thus, could affect the expression of target proteins. We used label-free quantitative (LFQ) proteomics to compare the profiles between patients with SCZ and CTRL subjects. Data from a subset of age-matched and gender-uniform participants (*n* = 18) in this pilot study were analyzed. Biological pathway analysis revealed significant abundance differences in a number of pathways, most notably mitochondrial and immune system proteins and protein metabolism (Fig. 4A). Upon further analysis, we found that markers of mitochondrial function are decreased and markers of immune activity are increased in patients with SCZ (Fig. 4A and fig. S3), in line with reports that these two processes are dysregulated in SCZ (*41*–*48*).

### HSP60, an HSP involved in mitochondrial function and inflammatory responses, is increased in buccal swabs from patients with SCZ

We searched for proteins with genes that are known targets of SP4 (*16*) and exhibited at least a 20% change in abundance, as detected by LFQ proteomics (*n* = 8). Fourteen proteins were identified from the GWASTop357 list of the overrepresented SCZ-risk genes with potential SP4 target sites (*16*), including three HSPs (table S2). One of these SCZ-risk genes with potential SP4 target sites is the chaperonin HSP60. HSP60 plays a crucial role in mitochondrial function and immune signaling, both of which have been implicated in SCZ pathology (*43*, *49**–**53*). To further investigate whether HSP60 is a potential biomarker for SCZ, we analyzed lysates from a larger subset of gender and age matched samples (*n* = 18) by targeted mass spectrometry (tMS). While there was variation in protein abundance between samples, likely due to the heterogeneous pathology of SCZ (*54**–**56*), on average, HSP60 was more abundant in patients with SCZ (abundance fold change = 1.77, patient abundance values normalized to the average of the CTRLs) (Fig. 4B).

SP4, NOS1AP, and Rasd1 proteins were not detected in our samples subjected to liquid chromatography–MS (LC-MS). This could be due to multiple reasons. SP4 may not have been detected as we isolated cytosolic proteins using our method. In addition, although *NOS1AP* and *Rasd1* mRNA were detected in buccal cells, the protein levels may be below detection limits for LC-MS. Bottom-up proteomic analysis identifies proteins with abundances that span several orders of magnitude. It is expected that proteins of lower abundance will require enrichment to be identified.

### Higher buccal swab HSP60 protein abundance correlates with poorer performance on the AX-CPT, impaired delayed recall and retention, and increased severity of symptoms

To assess the relationship between HSP60 levels and visual contour integration, we performed correlation analysis. HSP60 abundance did not correlate with accuracy measurements or reaction times on the JOVI task (table S4). However, multiple linear regression analyses revealed a significant relationship between HSP60 abundance and reaction time on the no-background catch trials (step 1 adjusted *R*^2^ = 0.076; *P* = 0.986; step 2 *R*^2^ = 0.598; *P* = 0.012) of the JOVI task after controlling for demographic variables (age, race, ethnicity, and sex) (table S5). HSP60 abundance did not show statistically significant correlation with PANSS five-factor scores (table S4). A series of multiple linear regressions corroborated these results when controlling for demographic variables (table S5).

To determine whether HSP60 protein abundance correlates with aberrant cognitive control and goal maintenance commonly seen in SCZ [reviewed in (*57*)], we compared normalized HSP60 abundance values with AX-CPT performance. We observed a statistically significant, moderate positive correlation between HSP60 abundance and BX error rate [*r*(18) = 0.606, *P* = 0.015] (table S4 and Fig. 4C). In addition, we observed moderate-strong positive correlations between HSP60 abundance and reaction time in all trial types [AX: *r*(18) = 0.608, *P* = 0.015; AY: *r*(18) = 0.671, *P* = 0.009; BX: *r*(18) = 0.527, *P* = 0.040; BY: *r*(18) = 0.717, *P* < 0.007] (table S4 and Fig. 4C). In a set of multiple linear regression analyses, we tested the degree to which age, sex, ethnicity, and race contributed to these findings. We found that age, sex, ethnicity, and race accounted for a nonsignificant amount of variance in BX error rate, and reaction time on the AX-CPT task [BX error rate (step 1 adjusted *R*^2^ = −0.108; *P* = 0.724), AX reaction time (step 1 adjusted *R*^2^ = −0.047; *P* = 0.724), BX reaction time (step 1 adjusted *R*^2^ = −0.091; *P* = 0.724), AY reaction time (step 1 adjusted *R*^2^ = −0.050; *P* = 0.724), and BY reaction time (step 1 adjusted *R*^2^ = −0.070; *P* = 0.724)] (table S5). The addition of HSP60 abundance to the analysis accounted for a significant amount of variance in BX error rate (step 2 adjusted *R*^2^ = 0.220; *P* = 0.042), AX reaction time (step 2 adjusted *R*^2^ = 0.393; *P* = 0.013), AY reaction time (step 2 adjusted *R*^2^ = 0.552; *P* = 0.003), and BY reaction time (step 2 adjusted *R*^2^ = 0.589; *P* = 0.003) (table S5). However, HSP60 abundance did not account for significant variance in BX reaction time (step 2 adjusted *R*^2^ = 0.132; *P* = 0.069). Despite showing a nonsignificant correlation between HSP60 and AX error rate [*r*(18) = 0.480; *P* = 0.058], regression analysis showed that while demographic covariates failed to account for significant variation (step 1 adjusted *R*^2^ = −0.047; *P* = 0.724), HSP60 abundance accounted for significant variation (step 2 *R*^2^ = 0.393; *P* = 0.013) (table S5).

HSP60 abundance did not significantly correlate with total recall [*r*(18) = −0.410, *P* = 0.121], delayed recall [*r*(18) = −0.484, *P* = 0.085], retention [*r*(18) = −0.483, *P* = 0.085], or recognition determination [*r*(18) = −0.119, *P* = 0.637] on the HVLT-R (table S4 and Fig. 4D). We then assessed these relationships, controlling for demographic variables (age, sex, ethnicity, and race), using a series of multiple linear regression analyses (table S5). All significant findings are reported here after analysis with the Benjamini-Hochberg procedure to correct for the false discovery rate in correlation and multiple linear regression analyses. Linear regression analyses revealed that demographic variables accounted for nonsignificant variance in total recall (step 1 adjusted *R*^2^ = −0.137; *P* = 0.910), delayed recall (step 1 adjusted *R*^2^ = −0.170; *P* = 0.910), and retention (step 1 adjusted *R*^2^ = −0.164; *P* = 0.910) in the HVLT-R, but the addition of HSP60 abundance in the analysis accounted for a significant amount of variance [total recall (step 2 adjusted *R*^2^ = 0.197; *P* = 0.041), delayed recall (step 2 adjusted *R*^2^ = 0.131; *P* = 0.041), and retention (step 2 adjusted *R*^2^ = 0.228; *P* = 0.041)] (table S5). Together, these data add additional support for the use of HSP60 protein abundance, in combination with *Sp4* mRNA, in buccal swabs as an indicator of processes involved in SCZ.

## DISCUSSION

In the current study, we identified two biomarker candidates for SCZ in buccal cells. Patients with SCZ have significantly higher levels of *Sp4* mRNA and HSP60 protein in buccal swabs than do CTRL subjects. Higher *Sp4* expression positively correlates with deficits in recognition discrimination and symptom severity. Specifically, we observed a relationship between *Sp4* expression and the presence of positive and negative symptoms and excitement. Similarly, HSP60 abundance correlates with slowed responses and increased BX error rate on tests of working memory (AX-CPT). However, our results from the JOVI task do not indicate a consistent relationship between *Sp4* mRNA expression or HSP60 abundance and visual contour integration. In SCZ, different tasks can be correlated with different symptom types (*58*, *59*). The JOVI mainly correlates with disorganized symptoms but not with positive or negative symptoms (*58*), and it is a reliable, robust, and consistent test for patients with SCZ (*60*). An issue with studying correlates of disorganized symptoms when studying patients living in the community (i.e., who are not inpatients at the time of testing) is that disorganization levels are typically low in community-dwelling patients receiving treatment, and this leads to a restricted range of scores and reduced sensitivity to linear model analyses (*61*).

Together, our results provide a rationale for larger-scale studies of buccal cell *Sp4* mRNA and HSP60 protein in buccal swabs in SCZ, including studies of their specificity to diagnosis and/or alternative classification types (e.g., B/SNIP biotypes), cognitive impairment, functional impairment, and trait versus state sensitivity (*62*). We observed that half of the patients with clear symptoms have buccal cell *Sp4* mRNA levels in the range of CTRL. This is a consistent finding as other biomarkers for SCZ demonstrate a similar pattern [discussed in (*63*)]. This overlap is due to multiple factors. There is biological heterogeneity in the brains of patients (*64**–**66*), there is overlap in risk genes between SCZ and other neurocognitive disorders such as bipolar disorder (*67*), and symptoms between patients vary [reviewed in (*68*)]. It is for this reason that a combination of validated biomarkers may be needed, and our study presents two of a future set of biomarkers that may be used in combination for the diagnosis of SCZ in a subset of patients.

*SP4* is a particularly compelling biomarker candidate for SCZ. In 2020, The Schizophrenia Working Group of the Psychiatric Genomics Consortium conducted the largest genome-wide association study (GWAS) to date, and the Schizophrenia Exome Meta-Analysis (SCHEMA) Consortium released the largest exome sequencing project to date (*19*, *22*). Notably, only two genes, *SP4* and *GRIN2A*, were identified as top 10 risk genes by both the GWAS and the SCHEMA (*16*). Fine mapping performed in a subsequent study demonstrated that patients with SCZ have rare disruptive coding variants in *SP4* and other genes (*22*). SP4 protein is increased in postmortem hippocampi of patients (*69*), and potential SP4 target genes that contain GC boxes, where SP4 binding occurs, are overrepresented within genes identified to increase SCZ-risk (*16*). Furthermore, phosphorylation of SP4, which is inversely proportional to SP4 levels, is increased in the cerebellum of patients with SCZ (*70*).

SP4 is a nuclear protein and member of the SP1 transcription factor family, and it binds to GC boxes in the promoter region of target genes (*16*). SP4 contains a DNA-binding domain and an N-terminal glutamine-rich activation domain, and its activity can be repressed by the SP transcription family member SP3 (*71*). Hippocampal SP4 levels are negatively regulated by *N*-methyl-d-aspartate (NMDA) receptor activity (*69*). SP4 negatively regulates expression of neurotrophin-3, allowing for proper dendritogenesis in cerebellar granule neurons (*72*). SP4 also modulates expression of γ-aminobutyric acid receptor subunits (*73*) and AMPA receptor subunits (*74*). Previous studies reported that a reduction in SP4 expression results in learning and memory deficits and other brain and behavior abnormalities associated with SCZ (*16*). There is an abundance of SCZ-risk genes that contain a GC box in their promoter region, suggesting that SP4 may act as an upstream regulator of several other SCZ-risk genes (*16*). While most high-risk genes for SCZ are brain-specific, the fact that SP4 is also expressed in epithelial cells (*23*) makes it a candidate as a buccal cell biomarker (*24*, *25*). Moreover, interleukin-6 (IL-6), a proinflammatory cytokine, and soluble IL-6 receptor (sIL-6R) signaling cascade can epigenetically activate SP4 expression in peritoneal tissue composed of endothelial cells (*25*).

SP4 plays a role in progression of esophageal squamous cell carcinoma and related cancers (*75*), and these carcinomas contain epithelial cells, which are similar to buccal cells. Anticancer agents target SP4 and other SP family members, and SP4 binds to promoter regions of genes that are involved in cellular proliferation, survival, and cell migration (*76*). SP4 is primarily expressed in neurons, specifically in brain, and it is expressed at low levels in buccal cells (*77*). Thus, it was expected that we did not detect SP4 protein in the buccal swabs.

The lack of correlation between the increased *Sp4* mRNA detected in buccal swabs of individuals with SCZ and the lack of corresponding SP4 protein changes in LFQ proteomics can be attributed to several factors. SP4 is a nuclear protein, and the protocol to generate protein lysate for LC-MS analysis avoids the use of detergents that are known to affect peptide resolution and suppress ionization. In addition, we did not detect SP4 by Western blot analysis (fig. S4) due to the fact that TRIzol extraction is not suitable for nuclear protein extraction. Therefore, it is possible that the generated postnuclear lysate excluded SP4. Moreover, posttranscriptional regulation and protein degradation can decouple mRNA and protein levels, while the sensitivity limitations of proteomics may fail to detect low-abundance proteins. In addition, biological variability among individuals and differences in temporal and spatial expression patterns can further complicate the correlation between mRNA and protein levels. These factors underscore the complexity of gene expression and the challenges in linking mRNA to protein abundance in different biological contexts.

We identified proteins associated with mitochondrial and immune pathways with altered abundance in our cohort. Recently, reports have emerged demonstrating mitochondrial dysfunction and chronic immune activation in patients with SCZ (*43*, *51*, *78**–**80*). These results are consistent with evidence of dysfunction in multiple cell types and neural pathways in SCZ (*43*, *78*, *81*). Mitochondrial dysfunction can lead to immune activation in the following manner; deficits in mitochondrial proteins lead to dysfunction of the electron transport chain and the production of oxidative and nitrosative stress responses (*43*). In turn, immune-inflammatory pathways are activated, resulting in chronic low-grade inflammation and neuroprogressive changes characteristic of SCZ (*43*).

We also identified HSP60 as a candidate buccal cell biomarker of SCZ in our pilot MS study. Previous reports support HSP60 as a biomarker for SCZ. Multiple studies, dating back to 1992, demonstrate that a subset of patients with SCZ have antibodies against HSP60 (*82**–**86*). HSP60 is present in mitochondria, and it preserves mitochondrial integrity, especially when cells are under stress (*87*). HSP60 has also been shown to regulate stem cells, allowing them to remain in an undifferentiated state (*88*). It is expressed at low levels in buccal cells (*77*), and this may be due to the fact that these cells are differentiated. Elevated levels of HSP60 in oral epithelial cells result in oral squamous cell carcinoma and high expression correlates with poor prognosis (*89*). The fact that high levels of SP4 and HSP60 result in oral cancers underscore the fact that HSP60 is a predicted target of SP4 as its proximal promoter contains two GC boxes (*16*, *33*, *34*).

Since HSP60 bridges the gap between mitochondrial function and immune response, both of which have been well documented in SCZ pathology (*43*, *51*, *52*, *78*), there is strong support for HSP60 as a biomarker for SCZ. HSP60, a multifaceted chaperonin, plays an important role in maintaining proteostasis in the mitochondria, and its elevated abundance is indicative of mitochondrial stress (*49*, *90*). Moreover, HSP60 can be secreted from cells, interact with immune cells via membrane-bound receptors (*91*), travel through the bloodstream, and affect distant sites (*91*). HSP60 plays an important role in several inflammatory and autoimmune diseases of the nervous system, including multiple sclerosis and myasthenia gravis (*91*). It also contributes to the pathogenesis of several neurological diseases, such as hereditary spastic paraplegias, MitCHAP-60, Alzheimer’s disease, Parkinson’s disease, and Huntington’s disease (*90*). Given that increased levels of buccal cell HSP60 may reflect a systemic immune-metabolic issue, HSP60 should be used as a biomarker of SCZ only in combination with other biomarkers, such as *Sp4* mRNA. Since HSP60 is ubiquitously expressed throughout the body (*50*), it is easy to collect and detect, as we show in buccal cell extracts. Additional studies, using a diverse and larger cohort, would further substantiate the association of HSP60 protein abundance increase with SCZ.

It is also notable that *Sp4* mRNA and HSP60 protein do not correlate with the same measures (table S4). Although HSP60 is predicted to be a downstream target of SP4, it is entirely possible that its up-regulation is independent of increased *Sp4* mRNA expression. SP4 is phosphorylated at serine-770, and this phosphorylation is regulated by NMDA receptor signaling, which in turn, modulates dendritogenesis (*92*). Lithium also regulates SP4 phosphorylation, which is impaired in neuropsychiatric disorders (*93*), and this phosphorylation regulates SP4 function. Thus, if the phosphorylation of the increased SP4 is not maintained at normal levels and cannot regulate downstream targets, the possibility exists that there may not be a direct mechanistic link between increased *Sp4* mRNA and HSP60 protein in our cohort. In addition, as part of a failed stress response, dysfunctional mitochondria in patients cause the buildup of *Sp4* mRNA, and this mRNA cannot be translated properly (*94*), and the increased *Sp4* mRNA would then not lead to increased HSP60 expression. Thus, although we chose HSP60 because of its predicted relationship to SP4, the mechanism by which it is increased in buccal cells of patients with SCZ may be SP4-independent.

While other biomarker candidates for SCZ have been reported using neuroimaging, cerebrospinal fluid, blood, fibroblasts, liver, and urine samples [reviewed in (*95*)], to the best of our knowledge, this study identifies biomarker candidates in buccal swabs. We chose buccal swabs as a potential source for peripheral biomarkers of SCZ for the following reasons. First, the CNS and buccal epithelium both develop from ectodermal tissue (*15*). The buccal epithelium is cellularly more homogenous than blood, making it a better source of material (*15*). Second, buccal epithelial cells contain a greater amount of disease-associated single-nucleotide polymorphisms and larger interindividual epigenetic variation than blood cells (*15*). Third, buccal swabs are far less invasive than methods used to acquire other commonly collected tissues, such as blood or cerebrospinal fluid. Additional support for the use of buccal cells as a source from biomarkers in brain disorders is that reduced telomere length in buccal cells correlates with Alzheimer’s disease (*96*). Furthermore, DNA methylation between buccal cells and the brain of patients with epilepsy demonstrates a correlation of *r* = 0.85 (*97*). Thus, cheek swabs are particularly appealing for the identification and exploration of biomarkers and, potentially, for clinical decision making. Any of these methods may show variation as SCZ is a heterogeneous disease and it is not uncommon to identify changes in only a subset of samples in any type of markers of SCZ (*98*, *99*). In our tMS experiments, we found that HSP60 varied in protein abundance between SCZ samples. In addition, the degree to which buccal cell biology truly reflects brain biology in the context of SCZ is still an active area of investigation.

While these data support the idea that *Sp4* mRNA expression and HSP60 may serve as buccal swab-based biomarkers for SCZ, our conclusions are limited by our sample size and cognitive battery. A larger sample size would allow us to validate our findings and explore differences between racial groups and gender. Implementing a more comprehensive cognitive battery could help to clarify our working memory findings and determine any relationships with cognitive deficits we did not explore, such as gain control. In addition, since our MS proteomics analysis only included cytosolic proteins, it would be beneficial to perform studies that include nuclear proteins, such as SP4. An important limitation of using buccal swabs is the limited amount of material that is obtained. The reduced amount of material, i.e., low protein yield, prevented robust antibody-based validation of our proteomics results. We performed Western blot using authenticated anti-HSP60 antibodies and human protein lysates and the determined linear range for the antibody. HSP60 protein in our samples was at the detection limit as assessed by Western blot analysis, although two of three samples from patients with SCZ demonstrated expression upon high contrast of the blot and no CTRL subjects showed expression (fig. S4). The commercially available antibody used to detect HSP60 for Western blot analysis is not optimized nor developed for clinical use.

Additional points to consider include our cohort size, especially our pilot MS with nine patients per group, and the fact that the study was limited to patients with SCZ and healthy CTRL patients. Future studies will include patients with bipolar disorder, other neurocognitive disorders, or neurodegenerative disorders to determine whether the biomarkers we identified are specific to SCZ, whether used individually or in combination. Furthermore, all of the patients in our study were taking their prescribed antipsychotic medications. There medications could have an effect on gene expression and inflammatory markers. Male mice chronically treated with haloperidol or olanzapine demonstrated changes to genes in medium spiny neurons, microglia, and astrocytes, and particularly those involved in mitochondrial function and inflammation (*100*). High concentrations of haloperidol activate macrophages (*101*), and glia play a role in cytokine response to antipsychotic medications (*102*). In humans, risperidone treatment increases immune system genes, while quetiapine and olanzapine do not (*103*). In contrast, antipsychotic treatment in macaques results in changes to genes in glucose homeostasis, mitochondria, and inflammation that are opposite to those seen in postmortem brain samples from patients with SCZ (*104*). Thus, it is possible that changes observed in our biological pathway analysis may be due, in part, to antipsychotic medication in patients with SCZ.

Together, our data suggest that *Sp4* mRNA expression and its downstream target HSP60 are potential biomarkers of SCZ. Our study reports buccal cell biomarkers that correlate with cognitive deficits observed in patients with SCZ. These findings have the potential to provide insight into disease pathophysiology and aid in the development of therapeutics. Replication of our findings in larger samples provides a foundation for future studies of the clinical utility of these and other buccal cell–based biological markers in SCZ research and clinical practice.

## MATERIALS AND METHODS

This study was approved by the Rutgers University Institutional Review Board under protocol Pro2020001322. All experiments were performed once except the RT-qPCR for *Sp4* mRNA for Origene primers due to limited buccal cell material.

### Participants

All subjects were between 18 and 65 years of age. Healthy CTRL subjects (*n* = 27) were recruited from Rutgers University and the surrounding community and met the following criteria: (i) no diagnosis of a neurological or psychiatric disorder; (ii) no history of psychotic episodes; (iii) not currently taking antipsychotics, antidepressants, or NMDA receptor agonists/antagonists; and (iv) no history of epilepsy or seizes. Patients with SCZ (*n* = 27) were recruited from the Rutgers University Behavioral Health Care acute, extended partial, and inpatient hospitalization programs. All subjects with SCZ who participated in the study met the following criteria: (i) had an SCZ or schizoaffective diagnosis confirmed with the SCID-5, (ii) no diagnosis of another neurological or psychiatric disorder (such as bipolar disorder or Parkinson’s disease), and (iii) no history of seizures or epilepsy. Patients with SCZ took their prescribed antipsychotic medications during the study. None of the patients with SCZ were in an inpatient program at the time of the study, but most patients had a history of at least one prior hospitalization due to psychosis. This study was approved by the Rutgers University Institutional Approval Board, Pro2020001322. Informed consent was obtained from all subjects.

### Structured interviews and cognitive tasks

Structured clinical interviews were performed at the Rutgers-Princeton CCNP on Rutgers University Busch campus in person or virtually over Zoom, based on the participant’s preference. All computerized cognitive tasks and neuropsychological measures were administered in-person at the CCNP.

### Structured clinical interview for the DSM-5

This 1- to 2-hour semistructured interview was used to determine any major DSM-5 diagnoses as described (*105*).

### SCI for the positive and negative syndrome scale

A 30-item structured interview was used to rate the intensity of psychotic symptoms, as well as general psychopathology (e.g., depression, anxiety, and mental status changes) in patients (*106*). Specifically, we used five-factor and cluster scoring to examine positive symptoms, negative symptoms, cognitive symptoms, excitement, depression, disorganization, anergia, thought disturbance, activation, and paranoia/belligerence.

### AX continuous performance task

This task was acquired from the Cognitive Neuroscience Test Reliability and Clinical applications for Schizophrenia (CNTRaCS) website by filling out the request form (*107*, *108*). This computerized cognitive task took 20 min to complete and made use of two buttons. Participants were instructed to press one button when the target stimulus (i.e., the letter “X”) was presented on the screen and the other button when any other stimulus was presented (i.e., the letter “A”). The order in which the stimuli were presented is important; the letter X is only a target when one specific letter precedes it (i.e., A➔X is a target, but B➔X is not). This task consisted of four blocks of 36 trials (144 total trials) (*108*). Specifically, this task consists of 104 AX trials, 16 AY trials, 16 BX trials, and 8 BY trials (*108*). Each trial consists of a cue, interstimulus interval, and target; each trial is separated by 1200 ms (*108*). The cue (i.e., A or B) is displayed for 1000 ms, the interstimulus interval lasts for 2000 ms, and the target (i.e., X or Y) is displayed for 500 ms with a 1500-ms response period (*108*).

### Jittered-orientation visual integration task

This task was acquired from CNTRaCS website by filling out the request form (*107*, *108*). This 15-min assessment of visual integration was conducted on a computer and made use of two buttons. Participants were asked to look at a series of images composed of Gabor elements in which a closed egg shape, embedded within randomly oriented Gabors, either pointed to the left or to the right (*37*). This task included six conditions in which the degree of orientational jitter of the egg’s contour elements was varied (0°, 7°–8°, 9°–10°, 11°–12°, 13°–14°, and 15–16°) (fig. S1A) (*108*). In addition, the task included some trials containing no background elements and others in which a curved line was drawn that connected the contour elements. These “catch” trials were included to identify subjects who responded randomly or were inattentive (fig. S1A). In total, the task consisted of 24 blocks (4 blocks per condition), each containing 12 trials (*108*). Each image was presented for 2 s with a 1-s interstimulus interval separating each trial (*37*).

### Hopkins Verbal Learning Test–Revised

This 30-min assessment of verbal learning and memory is composed of three learning trials, a delayed recall trial, and a recognition trial (*109*). In each learning trial, participants were read a list of 12 nouns (each word belongs to one of three semantic categories, such as “vegetables”) and asked to repeat the words out loud after the experimenter finished reading the list (*109*). The delayed recall trial was administered 20 to 25 min after the learning trials (*109*). During this portion of the task, participants were asked to verbally recall any words they remembered from the learning trials (*109*). The recognition trial was then administered. During the recognition trial, participants were read a list of words and indicated whether or not the word was included in the learning trials by responding “yes” or “no” (*109*).

### Buccal swabs

Two buccal swabs were collected from each participant during the third session after completion of the cognitive tasks. Subjects were instructed to not eat or drink for 2 hours before swabbing. Buccal swabbing was performed by rotating a Cytobrush GT (Cooper surgical, Trumbull, CT, USA) for protein and mRNA collection, on the inside of the subject’s cheek. Each cheek was swabbed for 1 min. The Cytobrush GT was inserted into a 15-ml conical tube containing ~4 ml of RNAlater (Thermo Fisher Scientific, Waltham, MA, USA, catalog no. AM7020). Both samples were stored at 4°C for 2 to 4 days after which samples were stored at −80°C until further processing.

### TRIzol extraction of RNA and protein from buccal cell samples

Samples were thawed on ice for an hour and then at room temperature for 15 min. RNAlater was removed via centrifugation at 5000*g* for 10 min at 4°C. Supernatant was removed and then samples were spun down for an additional 5 min at 5000*g* at 4°C. Next, RNA and protein were extracted from the samples using TRIzol (Thermo Fisher Scientific, catalog no. 15596026) following the manufacturer’s protocol.

### Real-time qPCR

Buccal cell mRNA was extracted as described above. cDNA was synthesized using the High-Capacity RNA-to-cDNA Kit (Applied Biosystems, Waltham, MA, USA, catalog no. 4387406) following the manufacturer’s protocol. RT-qPCR was performed using PowerUp SYBR Green Master Mix (Thermo Fisher Scientific, catalog no. A25780) and gene-specific primers (table S1). Samples were run in triplicate or quadruplicate in a 96-well plate (Thermo Fisher Scientific, catalog no. 4481190). Human brain cDNA (Zyagen, San Diego, CA, USA, catalog no. HD-201) was used as a positive control. QuantStudio 3 (Applied Biosystems) was used to determine threshold cycle (Ct) values for *SP4*, *Dexras1*, *NOS1AP*, and glyceraldehyde-3-phosphate dehydrogenase (*GAPDH*) expression*.* Relative gene expression was determined by normalizing to *GAPDH* and performing 2^(–ΔCt)^ calculations following the methodology outlined in Schmittgen and Livak (*110*).

### Proteomic analysis

LFQ proteomics was performed on extracts from a subset (males, ages 22 to 51) of patients with SCZ (*n* = 9) and age- and gender-matched CTRL subjects (*n* = 9). We only performed MS analysis on a subset of samples because only this subset had enough protein for both MS and Western blot analysis. MS analysis was performed by the Integrated Mass Spectrometry Unit at Michigan State University with blinded samples. Five micrograms of protein was buffer exchanged with 25 mM ammonium bicarbonate (AMBIC) using spin filtration (3 kDa MWCO). Detergents were removed via four rounds of ethyl acetate extraction (*111*). Samples were dried to completion and resuspended in digestion buffer [25 mM AMBIC/50% acetonitrile (ACN)] with 500 ng of rLys-C (Promega, catalog no. V1671) and 1 μg of trypsin (Promega, catalog no. V5280). The samples were digested overnight at 37°C. The digestion solution was transferred to clean tubes and dried completely at 30°C. Last, the samples were resuspended in 25 μl of 2% ACN and 0.1% formic acid (FA).

nanoLC–tandem MS separations were performed with a Thermo Fisher Scientific Ultimate 3000 RSLCnano System. Peptides were desalted in-line using a 3-μm-diameter bead C18 column (75 μm by 20 mm) with 2% ACN and 0.1% FA for 8.75 min with a flow rate of 2 μl/min at 40°C. The trap column was then brought in-line with a 2-μm-diameter bead, C18 EASY-Spray column (75 μm by 250 mm) for analytical separation over 127.5 min with a flow rate of 350 nl/min at 40°C. The mobile phase consisted of 0.1% FA (buffer A) and 0.1% FA in ACN (buffer B). The separation gradient was as follows: 8.75 min desalting, 98.75 min 4 to 40% B, 2 min 40 to 65% B, 3 min 65 to 95% B, 11 min 95% B, 1 min 95 to 4% B, 3 min 4% B. Five microliters of each sample was injected.

As previously reported (*112*), top 20 data-dependent MS analysis was performed with a Q Exactive HF-X Hybrid Quadrupole-Orbitrap Mass Spectrometer. MS1 resolution was 60 K at 200 mass/charge ratio (*m*/*z*) with a maximum injection time of 45 ms, AGC target of 3E6, and scan range of 300 to 1500 *m*/*z*. MS2 resolution was 30 K at 200 *m*/*z*, with a maximum injection time of 54 ms, AGC target of 1 × 10^5^, and isolation range of 1.3 *m*/*z*. HCD normalized collision energy was 28. Only ions with charge states from +2 to +6 were selected for fragmentation, and dynamic exclusion was set to 30 s. The electrospray voltage was 1.9 kV at a 2.0-mm tip to inlet distance. The ion capillary temperature was 280°C, and the radio frequency level was 55.0. All other parameters were set as default.

Protein identification was conducted by Proteome Discoverer Software version 2.5.0.400. Spectra were searched with Sequest against the combined reviewed *Homo sapiens* UniProt protein database (UP000005460), including L0R552, contaminant sequences, trypsin (Acc: P00761), and LysC (Acc:Q02SZ7). Enzyme specificity was set to trypsin with an MS1 tolerance of 10 parts per million and a fragment tolerance of 0.02 Da. Oxidation (M), acetylation (protein N-term), and methionine loss (protein N-term) were set as dynamic modifications. False discovery rates were set to 0.01 using the Percolator node. Two unique peptides were required for protein identification. Quantitative ratios were determined using the precursor ion quantitation node. This node calculates the abundance of a peptide as the summation of its quantitative peptide spectral matches. Abundances values were normalized to the total peptide amount of *H. sapiens* sequences. Protein ratios are determined by pairwise comparison.

For pathway analysis, proteins were uploaded into Ingenuity Pathway Analysis (IPA; QIAGEN Inc., Hilden, Germany) and filtered by removing proteins with log_2_ < −0.3 and log_2_ > 0.3. Data were analyzed with the use of QIAGEN IPA (QIAGEN Inc., https://digitalinsights.qiagen.com/IPA). For biomarker identification, LFQ was run on a subset (males, ages 24 to 41) of patients (*n* = 4) and matched CTRLs (*n* = 4) and filtered by removing proteins with fewer than four peptide spectral matches across all samples, common contaminant proteins, and proteins with less than a 20% difference between groups. This list of proteins was then compared to a list of potential SP4 target genes (*16*). Fourteen possible protein biomarkers were identified (table S2), and HSP60 was chosen for targeted quantitation (tMS) in a larger subset of gender- and age-matched samples (males, ages 22 to 51, *n* = 18).

tMS analysis used the sample preparation and separation protocols described above. Targeted peptide sequences and ions are reported in (table S3). MS1 resolution was 120 K at 200 *m*/*z* with a maximum injection time of 5 ms, AGC target of 3 × 10^6^, and scan range of 300 to 1500 *m*/*z*. MS2 resolution was 30 K at 200 *m*/*z*, with a maximum injection time of 54 ms, AGC target of 2 × 10^5^, and isolation range of 1.6 *m*/*z*. HCD normalized collision energy was 28. Skyline v. 23.1.0 was used for fragment ion quantitation. Peak retention times were manually validated. The peak areas of the top 5 to 11 fragment ions of each peptide were used for quantitation. Fragment spectra were validated by comparison to the Human NIST HCD Selected library. Peptide abundance was normalized by performing a separate, data-dependent tandem mass spectrometry (ddMS) experiment (using the parameters reported above) for each sample and extracting the “total peptide amount” normalization factor.

### Statistical analysis

GraphPad Prism 10 and IBM SPSS Statistics (Version 29.0.2.0) software were used to analyze all data. Correlations or differences between groups were considered significant when *P* < 0.05. The Benjamini-Hochberg procedure was used to correct for the false discovery rate in correlation and multiple linear regression analyses. We recruited CTRL subjects with similar anthropometric data (age, race, and gender) to patients with SCZ to maintain equivalent demographics between the groups (*35*). All data represent means ± SD.
