# Supplementary material for: Differential SP4 expression and HSP60 abundance in buccal swabs from patients with schizophrenia
Source: Sci Adv. 2026 Mar 4;12(10):eaeb0460. doi: 10.1126/sciadv.aeb0460 (PMC12959401; doi:10.1126/sciadv.aeb0460)
Supplement: Supplementary file 1 — Figs. S1 to S4 Tables S1 to S5 Legend for data S1 [file sciadv.aeb0460_sm.pdf]

Science Advances
AAAS

Supplementary Materials for

Differential *SP4* expression and HSP60 abundance in buccal swabs from patients with schizophrenia

Christen M. Crosta *et al.*

Corresponding author: Bonnie L. Firestein, bonnie.firestein@uky.edu

Sci. Adv. **12**, eaeb0460 (2026)
DOI: 10.1126/sciadv.aeb0460

The PDF file includes:

Figs. S1 to S4
Tables S1 to S5
Legend for data S1

Other Supplementary Material for this manuscript includes the following:

Data S1

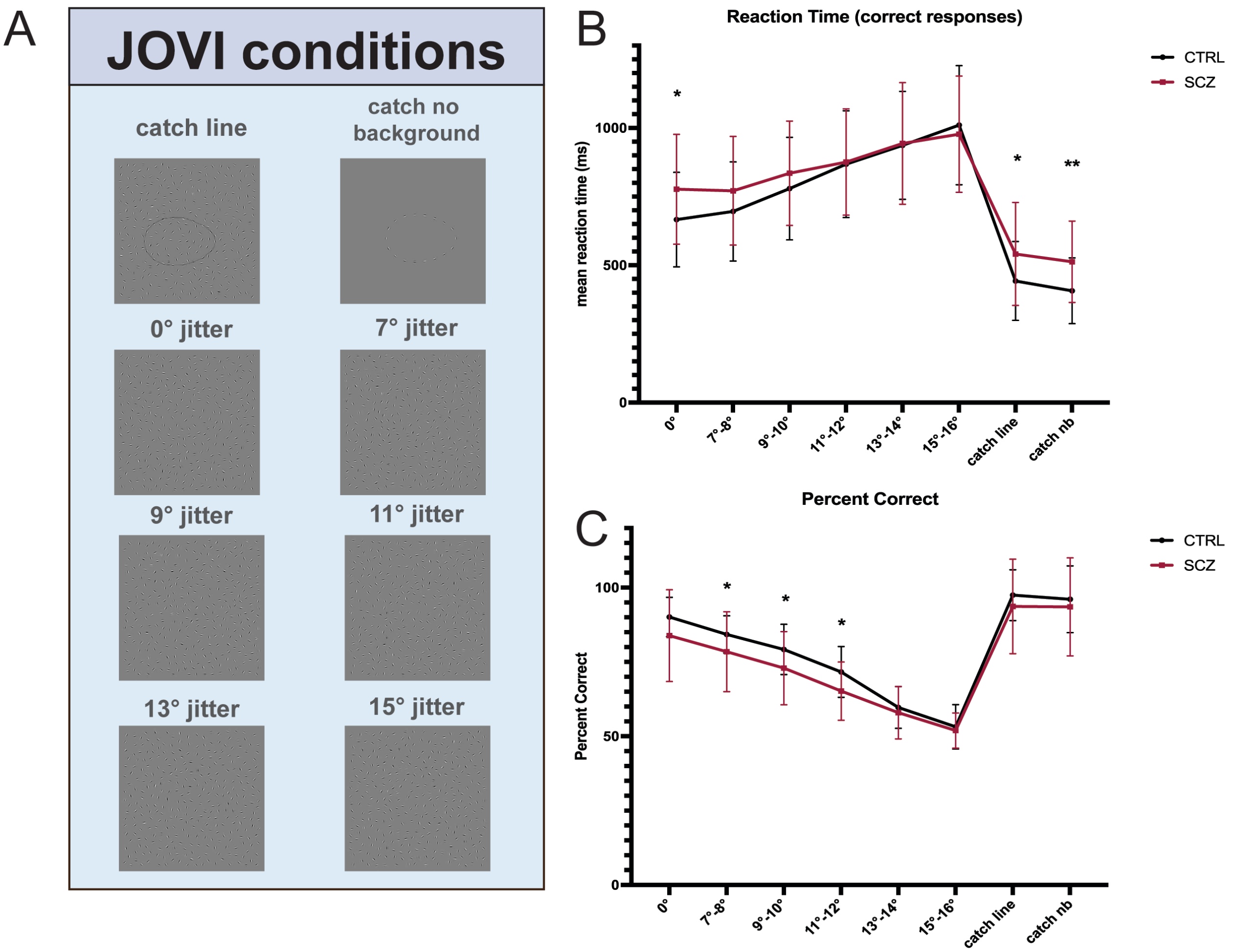

Fig. S1. Correlational analyses suggest that overall, *SP4* mRNA expression and HSP60 abundance in buccal swabs are not correlated to contour integration. (A) Examples of the 8 trial types in the JOVI task. **(B)** Mean reaction time by trial type. There was a statistically significant difference in JOVI reaction time between groups for 0°, catch line, and catch no background, Welch's $F(1, 50.85) = 4.75$, $p = 0.034$; $F(1, 48.67) = 4.68$, $p = 0.035$; $F(1, 48.06) = 8.09$, $p = 0.007$. **(C)** Mean percent correct by trial type. The was a statistically significant difference in JOVI accuracy between groups for 7°, 9°, and 11°, Welch's $F(1, 36.77) = 4.18$, $p = 0.048$; $F(1, 46.10) = 4.80$, $p = 0.034$; $F(1, 51.04) = 6.64$, $p = 0.013$.

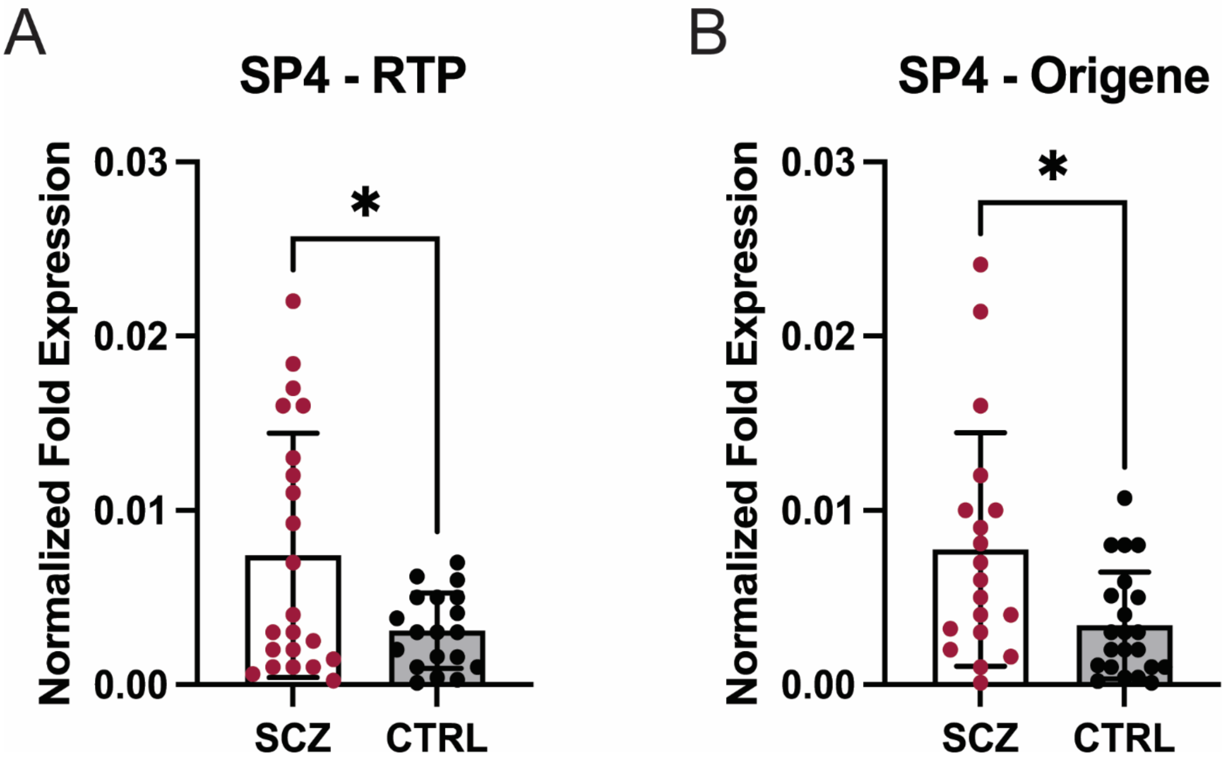

Fig. S2. Expression of *SP4* mRNA is increased in buccal cells of patients with SCZ in an orthogonal primer set. (A) RT-qPCR analysis of *SP4* mRNA (normalized to *GAPDH*) using an orthogonal primer set from RealTimePrimers in buccal cell extracts from patients with schizophrenia (SCZ; n=22) or control subjects (CTRL; n=19). *p=0.0109 as determined by unpaired t-test with Welch's correction for unequal variances. **(B)** RT-qPCR analysis of *SP4* mRNA (normalized to *GAPDH*) using the Origene primer set from Fig. 1 in buccal cell extracts from patients with SCZ (n=19) or CTRL subjects(n=22). *p=0.0153 as determined by unpaired t-test with Welch's correction for unequal variances. Error bars represent SD. For all analyses, outliers were removed (A=7, B=4) using the ROUT method (Q=1%), and samples with Ct values below detection levels or without an age-, race-, and gender-matched control were removed.

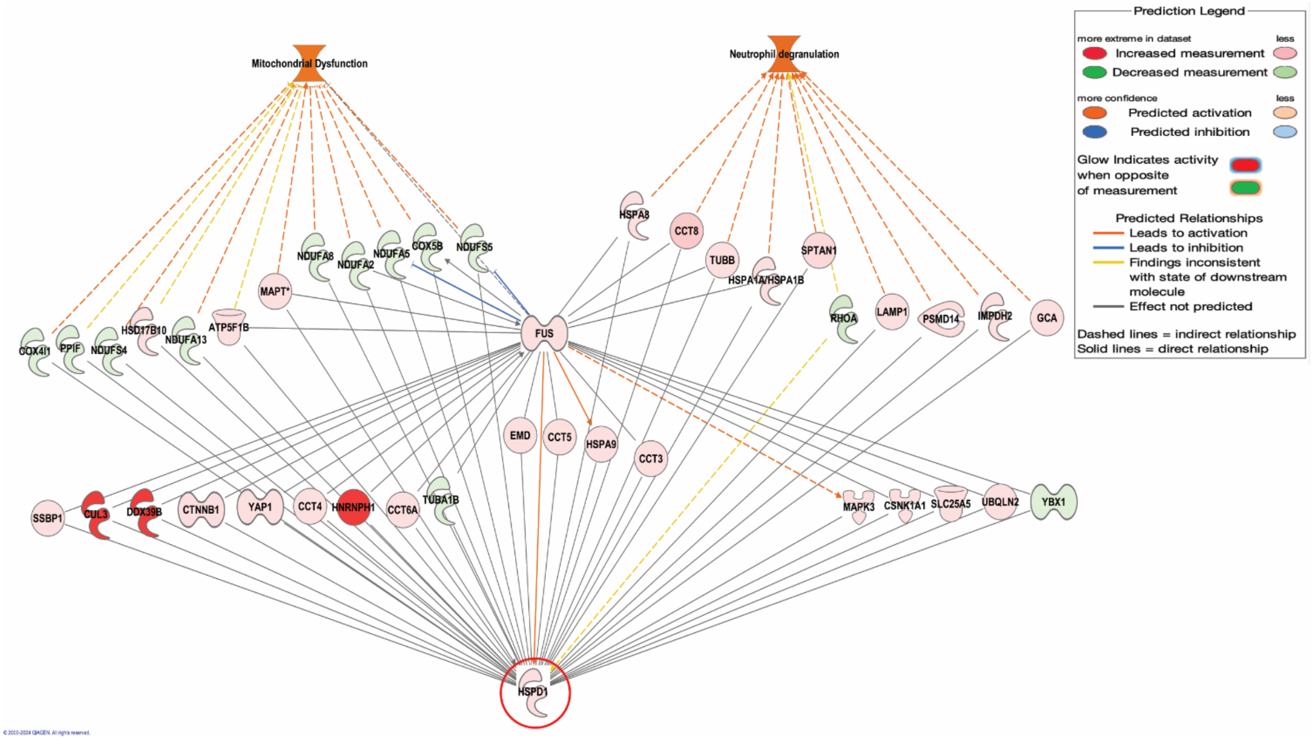

Fig. S3. HSP60 and related protein abundance in buccal swabs and predicted involvement in mitochondrial and neutrophil degranulation pathways. LFQ proteomics was used to reveal differences in the abundance of proteins in buccal swabs of SCZ patients and control subjects. Schematic of the involvement of HSP60 (HSPD1), which is higher in swabs from SCZ patients, in mitochondrial dysfunction pathways (left) and neutrophil degranulation (right). Red indicates pathway proteins that are increased in abundance in samples from patients with SCZ. Green indicates pathway proteins that are decreased in abundance in samples from patients with SCZ. Orange indicates predicted activation, and blue indicates predicted inhibition. The network and image were generated through the use of QIAGEN IPA (QIAGEN Inc., https://digitalinsights.qiagen.com/IPA).

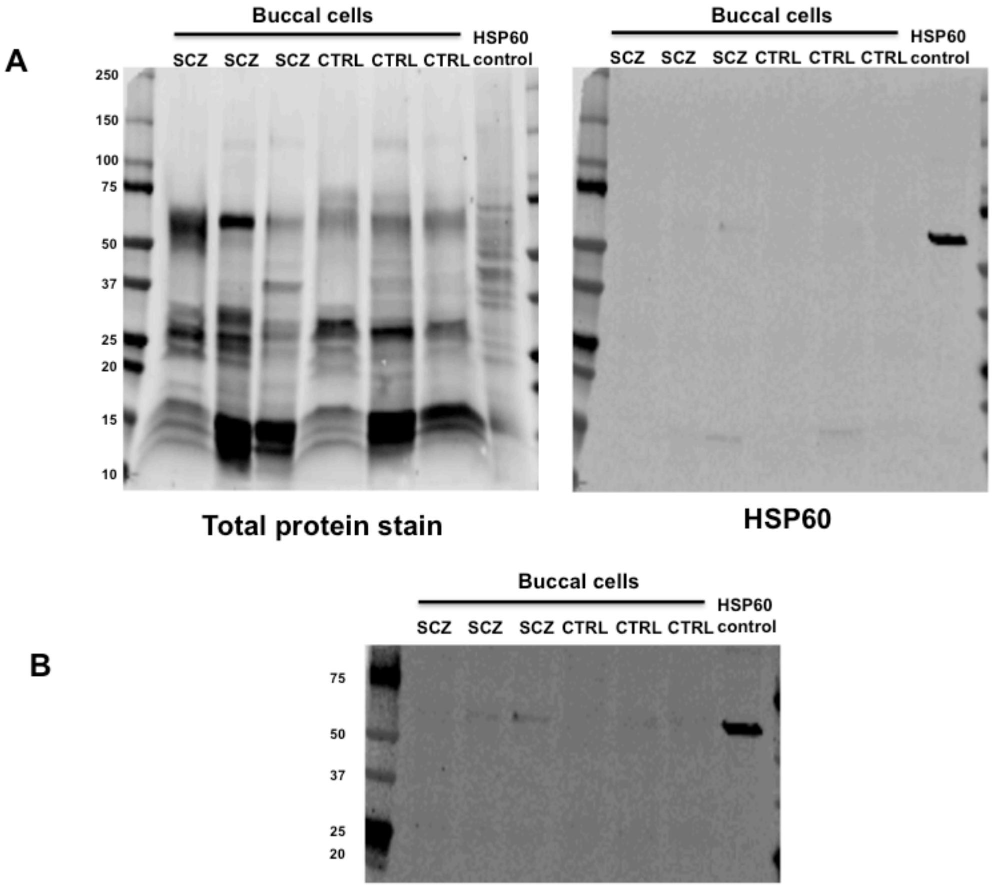

Fig. S4. Expression of HSP60 is detected in buccal cells of patients with SCZ. (A) Buccal cell extracts (5μg) from patients with schizophrenia (SCZ) or control subjects (CTRL) were resolved by SDS-PAGE, followed by REVERT total protein stain and Western blot analysis of HSP60 protein (anti-HSP60; Cell Signaling, cat no. 12165). Faint band were observed at 60 kDa in two of the three SCZ extracts and none of the CTRL extracts. **(B)** Intensity of the blot was increased to show bands present in the second and third SCZ sample. HSP60 control = human brain lysate from cognitively unimpaired subject.

Table S1. Primers used for RT-qPCR reactions.

Gene name	Company	Cat. Number	Forward	Reverse
GAPDH	IDT Technologies	25 nmole DNA Oligo	AGCCGCATCTTCTTTTGCGT	CGCCCAATACGACCAAATCCG
CAPON (NOS1AP) Human	Origene	HP211078	AGAGACACCGCTGTCCACTCAC	AGCCAACTGGTCCTTCAGCAAG
Dexras1 (RASD1) Human	Origene	HP211967	CACCGCAAGTTCTACTCCATCC	GGTTGTCCAGACTGAACACCAG
Transcription factor Sp4 (SP4) Human	Origene	HP206694	CGAAGAGTTGCCTGTTCCTGTC	CAGTATGCCAGCGAAGATGTGC
Human Sp4 transcription factor (SP4)	RealTimePrimers	VHPS-8770	CAGTATGCAAGCACATCAGC	GTGGTGGAATAGCCTGAATG

Table S2. SP4 targets that are SCZ risk genes.

Gene Symbol	Protein	UniProt accession number
YWHAE	14-3-3 Epsilon	P62258
NDUFA2	NADH:Ubiquinone Oxidoreductase Subunit A2	O43678
HSPE1	Heat Shock Protein Family E (Hsp10) Member 1	P61604
PSMA4	Proteasome 20S Subunit Alpha 4	P25789
RPL13	Ribosomal Protein L13	P26373
PDIA3	Protein Disulfide Isomerase Family A Member 3	P30101
ARHGAP1	Rho GTPase Activating Protein 1	Q07960
HSPD1	Heat Shock Protein Family D (Hsp60) Member 1	P10809
HMOX2	Heme Oxygenase 2	P30519
CTNND1	Catenin Delta 1	O60716
CLU	Clusterin	P10909
HSPA9	Heat Shock Protein Family A (Hsp70) Member 9	P38646
SEC11A	SEC11 Homolog A, Signal Peptidase Complex Subunit	P67812
MAPT	Microtubule Associated Protein Tau	P10636-5

Table S3. HSP60 tMS peptides.

Sequence	Charge	Precursor (m/z)
LVQDVANNTNEEAGDGTTTATVLAR	3	854.0877
VGEVIVTK	2	422.7606
NAGVEGSLIVEK	2	608.3326

Table S4. Pearson's correlations with Benjamini-Hochberg correction for proposed buccal cell biomarkers (*Sp4* mRNA and HSP60 abundance) and markers of cognitive performance and schizophrenia symptomology. Significant values are in bold.

Correlations

		Sp4 mRNA	HSP60
AX-CPT, AX error rate	Pearson's r	-0.114	0.480
	p-value (2-tailed)	0.704	0.058
	N	43	18
AX-CPT, AY error rate	Pearson's r	0.068	0.327
	p-value (2-tailed)	0.761	0.211
	N	43	18
AX-CPT, BX error rate	Pearson's r	0.099	**0.606***
	p-value (2-tailed)	0.704	**0.015**
	N	43	18
AX-CPT, BY error rate	Pearson's r	0.030	0.301
	p-value (2-tailed)	0.848	0.225
	N	43	18
AX-CPT, AX reaction time	Pearson's r	0.226	**0.608***
	p-value (2-tailed)	0.289	**0.015**
	N	43	18
AX-CPT, AY reaction time	Pearson's r	0.316	**0.671****
	p-value (2-tailed)	0.103	**0.009**
	N	43	18
AX-CPT, BX reaction time	Pearson's r	0.400	**0.527***
	p-value (2-tailed)	0.062	**0.040**
	N	43	18
AX-CPT, BY reaction time	Pearson's r	0.352	**0.717****
	p-value (2-tailed)	0.082	**0.007**
	N	43	18
HVLT-R, total recall	Pearson's r	-0.124	-0.410
	p-value (2-tailed)	0.555	0.121

	N	45	18
HVLT-R, delayed recall	Pearson's r	-0.135	-0.484
	p-value (2-tailed)	0.555	0.085
	N	45	18
HVLT-R, retention	Pearson's r	-0.005	-0.483
	p-value (2-tailed)	0.973	0.085
	N	45	18
HLVT-R, recognition determination	Pearson's r	**-0.431***	-0.119
	p-value (2-tailed)	**0.012**	0.637
	N	45	18
PANSS five-factor, Positive	Pearson's r	**0.393***	0.335
	p-value (2-tailed)	**0.026**	0.217
	N	45	18
PANSS five-factor, Negative	Pearson's r	**0.329***	0.579
	p-value (2-tailed)	**0.046**	0.059
	N	45	18
PANSS five-factor, Cognitive	Pearson's r	0.079	0.416
	p-value (2-tailed)	0.605	0.144
	N	45	18
PANSS five-factor, Excitement	Pearson's r	**0.379***	0.462
	p-value (2-tailed)	**0.026**	0.134
	N	45	18
PANSS five-factor, Depression	Pearson's r	0.097	0.245
	p-value (2-tailed)	0.605	0.327
	N	45	18
JOVI, 0° accuracy	Pearson's r	0.170	-0.163
	p-value (2-tailed)	0.672	0.636
	N	45	18
JOVI, 7-8° accuracy	Pearson's r	0.133	-0.339
	p-value (2-tailed)	0.672	0.451
	N	45	18
JOVI, 9-10° accuracy	Pearson's r	0.182	-0.283

	p-value (2-tailed)	0.672	0.516
	N	45	18
JOVI, 11-12° accuracy	Pearson's r	0.092	-0.390
	p-value (2-tailed)	0.672	0.351
	N	45	18
JOVI, 13-14° accuracy	Pearson's r	-0.013	-0.129
	p-value (2-tailed)	0.930	0.697
	N	45	18
JOVI, 15-16° accuracy	Pearson's r	0.367	-0.189
	p-value (2-tailed)	0.208	0.636
	N	45	18
JOVI, line catch accuracy	Pearson's r	0.223	0.000
	p-value (2-tailed)	0.672	0.999
	N	45	18
JOVI, no background catch accuracy	Pearson's r	0.205	-0.261
	p-value (2-tailed)	0.672	0.516
	N	45	18
JOVI, 0° reaction time	Pearson's r	0.146	0.399
	p-value (2-tailed)	0.672	0.351
	N	45	18
JOVI, 7-8° reaction time	Pearson's r	0.099	0.418
	p-value (2-tailed)	0.672	0.351
	N	45	18
JOVI, 9-10° reaction time	Pearson's r	0.100	0.164
	p-value (2-tailed)	0.672	0.636
	N	45	18
JOVI, 11-12° reaction time	Pearson's r	0.060	0.054
	p-value (2-tailed)	0.742	0.886
	N	45	18
JOVI, 13-14° reaction time	Pearson's r	0.099	-0.247
	p-value (2-tailed)	0.672	0.516
	N	45	18
JOVI, 15-16° reaction time	Pearson's r	0.107	-0.524
	p-value (2-tailed)	0.672	0.205
	N	45	18
JOVI, line catch reaction time	Pearson's r	0.061	0.281
	p-value (2-tailed)	0.742	0.516
	N	45	18

JOVI, no background catch reaction time	Pearson's r	0.168	0.652
	p-value (2-tailed)	0.672	0.054
	N	44	18

*. Correlation is significant at the 0.05 level (2-tailed).

**. Correlation is significant at the 0.01 level (2-tailed).

Table S5. Multiple linear regression analyses using a hierarchal method. The impact of demographic covariates (age, sex, ethnicity, and race) on cognitive performance and sympotomology variance were tested in the first block (Step 1), and the impact of the proposed biomarkers were measured in the second block (Step 2). All p-values corrected via the Benjamini Hochberg procedure. Significant values are in bold.

Independent Variable	Dependent Variable	Adjusted R^2 (Step 1)	*p*-value (Step 1)	Adjusted R^2 (Step 2)	*p*-value (Step 2)
Sp4	AX-CPT, AX error rate	0.033	0.447	0.018	0.699
Sp4	AX-CPT, BX error rate	0.033	0.927	0.019	0.699
Sp4	AX-CPT, AY error rate	0.16	0.447	0.138	0.927
Sp4	AX-CPT, BY error rate	0.022	0.447	-0.005	0.927
Sp4	AX-CPT, AX reaction time	0.017	0.447	0.047	0.288
Sp4	AX-CPT, BX reaction time	-0.012	0.490	**0.185**	**0.024**
Sp4	AX-CPT, AY reaction time	0.048	0.447	0.136	0.088
Sp4	AX-CPT, BY reaction time	-0.003	0.490	0.107	0.088
Sp4	HVLT-R, total recall	0.048	0.572	0.032	0.764
Sp4	HVLT-R, delayed recall	0.016	0.572	0.003	0.764
Sp4	HVLT-R, retention	-0.021	0.572	-0.047	0.903
Sp4	HLVT-R, recognition determination	-0.024	0.572	**0.129**	**0.028**
Sp4	PANSS five-factor, Positive	0.023	0.750	**0.168**	**0.018**
Sp4	PANSS five-factor, Negative	-0.043	0.835	0.054	0.050
Sp4	PANSS five-factor, Cognitive	0.042	0.75	0.03	0.473
Sp4	PANSS five-factor, Excitement	-0.023	0.835	**0.137**	**0.018**
Sp4	PANSS five-factor, Depression	-0.062	0.835	-0.071	0.473
Sp4	JOVI, 0° accuracy	0.030	0.606	0.022	0.842
Sp4	JOVI, 7-8° accuracy	-0.005	0.606	0.013	0.842
Sp4	JOVI, 9-10° accuracy	-0.005	0.606	0.008	0.842
Sp4	JOVI, 11-12° accuracy	-0.022	0.606	0.042	0.842
Sp4	JOVI, 13-14° accuracy	-0.072	0.899	-0.098	0.868
Sp4	JOVI, 15-16° accuracy	-0.012	0.606	0.093	0.368
Sp4	JOVI, line catch accuracy	0.016	0.606	0.051	0.693
Sp4	JOVI, no background catch accuracy	0.048	0.606	0.08	0.693

Sp4	JOVI, 0° reaction time	0.099	0.606		0.105	0.842
Sp4	JOVI, 7-8° reaction time	0.151	0.496		0.138	0.842
Sp4	JOVI, 9-10° reaction time	0.012	0.606		-0.004	0.842
Sp4	JOVI, 11-12° reaction time	-0.014	0.606		-0.039	0.868
Sp4	JOVI, 13-14° reaction time	-0.008	0.606		-0.032	0.868
Sp4	JOVI, 15-16° reaction time	-0.024	0.606		-0.046	0.842
Sp4	JOVI, line catch reaction time	0.080	0.606		0.062	0.842
Sp4	JOVI, no background catch reaction time	0.046	0.606		0.055	0.842
HSP60	AX-CPT, AX error rate	0.223	0.364		**0.427**	**0.046**
HSP60	AX-CPT, BX error rate	-0.108	0.724		**0.220**	**0.042**
HSP60	AX-CPT, AY error rate	0.309	0.344		0.312	0.370
HSP60	AX-CPT, BY error rate	-0.005	0.724		-0.053	0.560
HSP60	AX-CPT, AX reaction time	-0.047	0.724		**0.393**	**0.013**
HSP60	AX-CPT, BX reaction time	-0.091	0.724		0.132	0.069
HSP60	AX-CPT, AY reaction time	-0.050	0.724		**0.552**	**0.003**
HSP60	AX-CPT, BY reaction time	-0.070	0.724		**0.589**	**0.003**
HSP60	HVLT-R, total recall	-0.137	0.910		**0.197**	**0.041**
HSP60	HVLT-R, delayed recall	-0.170	0.910		**0.131**	**0.041**
HSP60	HVLT-R retention	-0.164	0.910		**0.228**	**0.041**
HSP60	HLVT-R recognition determination	-0.158	0.910		-0.234	0.715
HSP60	PANSS five-factor, Positive	-0.050	0.909		0.048	0.211
HSP60	PANSS five-factor, Negative	-0.170	0.909		0.241	0.060
HSP60	PANSS five-factor, Cognitive	-0.147	0.909		0.057	0.165
HSP60	PANSS five-factor, Excitement	0.038	0.909		0.109	0.211
HSP60	PANSS five-factor, Depression	-0.103	0.909		-0.073	0.258
HSP60	JOVI, 0° accuracy	0.025	0.986		0.001	0.662
HSP60	JOVI, 7-8° accuracy	-0.064	0.986		0.007	0.414
HSP60	JOVI, 9-10° accuracy	0.070	0.986		0.042	0.662
HSP60	JOVI, 11-12° accuracy	-0.111	0.986		0.025	0.293

HSP60	JOVI, 13-14° accuracy	-0.061	0.986		0.125	0.762
HSP60	JOVI, 15-16° accuracy	-0.011	0.986		-0.085	0.834
HSP60	JOVI, line catch accuracy	-0.134	0.986		-0.193	0.731
HSP60	JOVI, no background catch accuracy	0.405	0.256		0.506	0.293
HSP60	JOVI, 0° reaction time	-0.124	0.986		0.046	0.293
HSP60	JOVI, 7-8° reaction time	-0.094	0.986		0.054	0.293
HSP60	JOVI, 9-10° reaction time	-0.177	0.986		-0.228	0.703
HSP60	JOVI, 11-12° reaction time	-0.202	0.986		-0.286	0.828
HSP60	JOVI, 13-14° reaction time	-0.189	0.986		-0.221	0.662
HSP60	JOVI, 15-16° reaction time	-0.144	0.986		0.075	0.293
HSP60	JOVI, line catch reaction time	-0.105	0.986		-0.120	0.662
HSP60	JOVI, no background catch reaction time	0.076	0.986		**0.598**	**0.012**

Data S1. Protein IDs from Proteomics Analysis. (separate file)
